# New and revised maimetshid wasps from Cretaceous ambers (Hymenoptera, Maimetshidae)

**DOI:** 10.3897/zookeys.130.1453

**Published:** 2011-09-24

**Authors:** Vincent Perrichot, Jaime Ortega-Blanco, Ryan C. McKellar, Xavier Delclòs, Dany Azar, André Nel, Paul Tafforeau, Michael S. Engel

**Affiliations:** 1 CNRS UMR 6118 Géosciences and Observatoire des Sciences de l’Univers de Rennes, Université Rennes 1, Campus de Beaulieu bât. 15, 263 avenue du Général Leclerc, 35042 Rennes Cedex, France; 2 Biodiversity Institute, University of Kansas, Lawrence, Kansas 66045, USA; 3 Departament d’Estratigrafia, Paleontologia i Geociències Marines, Facultat de Geologia, Universitat de Barcelona, Martí i Franqués s/n, 08028 Barcelona, Spain; 4 Department of Earth and Atmospheric Sciences, 1-26 Earth Sciences Building, University of Alberta, Edmonton, Alberta T6G 2E3, Canada; 5 Lebanese University, Faculty of Sciences II, Department of Biology, Fanar Matn P.O. Box 26110217, Lebanon; 6 CNRS UMR 7205, Muséum National d’Histoire Naturelle, CP 50, Entomologie, 45 rue Buffon, F-75005, Paris, France; 7 European Synchrotron Radiation Facility, BP 220, 6 rue Jules Horowitz, 38043 Grenoble, France; 8 Division of Entomology (Paleoentomology), Natural History Museum, and Department of Ecology and Evolutionary Biology, 1501 Crestline Drive – Suite 140, University of Kansas, Lawrence, Kansas 66049-2811, USA; 9 Division of Invertebrate Zoology, American Museum of Natural History, Central Park West at 79th Street, New York, New York 10024-5192, USA

**Keywords:** Hymenoptera, Maimetshidae, France, Lebanon, Spain, Canada, Neocomian, Albian, Campanian, Mesozoic, taxonomy

## Abstract

New material of the wasp family Maimetshidae (Apocrita) is presented from four Cretaceous amber deposits – the Neocomian of Lebanon, the Early Albian of Spain, the latest Albian/earliest Cenomanian of France, and the Campanian of Canada. The new record from Canadian Cretaceous amber extends the temporal and paleogeographical range of the family. New material from France is assignable to *Guyotemaimetsha enigmatica* Perrichot et al. including the first females for the species, while a series of males and females from Spain are described and figured as *Iberomaimetsha* Ortega-Blanco, Perrichot & Engel, **gen. n.**, with the two new species *Iberomaimetsha rasnitsyni* Ortega-Blanco, Perrichot & Engel, **sp. n.** and *Iberomaimetsha nihtmara* Ortega-Blanco, Delclòs & Engel, **sp. n.**; a single female from Lebanon is described and figured as *Ahiromaimetsha najlae* Perrichot, Azar, Nel & Engel, **gen. et sp. n.**, and a single male from Canada is described and figured as *Ahstemiam cellula* McKellar & Engel, **gen. et sp. n.** The taxa are compared with other maimetshids, a key to genera and species is given, and brief comments made on the family.

## Dedication

We lovingly dedicate this humble contribution to the discoverer of the Maimetshidae, our friend and colleague, Prof. Alexandr P. Rasnitsyn, on the occasion of his 75^th^ birthday. It is greatly hoped that Alex, as the great docent of hymenopterological paleontology, will look upon our work with favor and pride, knowing that his rich body of inquiry has inspired every aspect of our own probes into the history of this fascinating order.

## Introduction

The family Maimetshidae was established as an extinct lineage of apocritan wasps based on a single incomplete female and male preserved in Santonian Taimyr amber (Rasnitsyn 1975). A single genus and species, *Maimetsha arctica* Rasnitsyn, was recognized and considered to be intermediate between Megalyridae and Ceraphronoidea, linking these otherwise disparate groups (Rasnitsyn 1975, 2002).

More than a quarter century later, Perrichot et al. (2004a) described a second genus and species which shared considerable similarities with *Maimetsha* Rasnitsyn and *Cretogonalys* Rasnitsyn, a genus originally attributed to Trigonalyidae but subsequently recognized as a possible maimetshid (Rasnitsyn and Brothers 2009). Given the uncertainties of the time, *Guyotemaimetsha* Perrichot et al. was considered as family *incertae sedis* (Perrichot et al. 2004a). Later, Perrichot (2009) reported new specimens of *Guyotemaimetsha* as well as undescribed material from Spanish amber, all of which appeared to suggest closer affinities with Trigonalyidae [This name often appears as Trigonalidae, given that Cresson (1887) first used this spelling. However, ICZN (1999: Art. 29.3) dictates that the name is to be based on the correct stem by deleting the case ending of the genitive singular, in the present case giving “Trigonalyd–”, thus the name would be automatically corrected to Trigonalydidae, with the same author and date, except that (Art. 29.3.1.1) notes that the unelided form, Trigonalyidae, is to be retained since the elided form (Trigonalydidae) is not in prevailing usage. Accordingly, Trigonalidae is a *nomen imperfectum*, which is *recte* Trigonalyidae Cresson, 1887].

Maimetshidae are presently known only from the Cretaceous and largely as inclusions in amber, similar in this respect to the families Radiophronidae (Ortega-Blanco et al. 2010), Stigmaphronidae (Rasnitsyn 1975, 1991; Engel and Grimaldi 2009; Ortega-Blanco et al. in press; McKellar and Engel in press), Alavarommatidae (Ortega-Blanco et al. 2011a), Gallorommatidae (Gibson et al. 2007; Ortega-Blanco et al. 2011a), and Serphitidae (Kozlov and Rasnitsyn 1979; McKellar and Engel 2011; Ortega-Blanco et al. 2011b; Engel et al. 2011). Rasnitsyn and Brothers (2009) described various maimetshids from Late Cretaceous compressions, and, as mentioned before, *Cretogonalys* is possibly also of this family, as could be the species of *Turgonalus* Rasnitsyn (Rasnitsyn 1990; Rasnitsyn et al. 1998).

The classification of Maimetshidae has been controversial, with various alternative interpretations of their putative relationships. For example, many authors have recognized them as an extinct, isolated family of Ceraphronoidea s.l. (Rasnitsyn 1975, 1977, 1988; Ronquist et al. 1999; Rasnitsyn and Brothers 2009; Perrichot 2009; Ortega-Blanco et al. 2010), while (Shaw (1988, 1990) considered them as a basal clade within the Megalyridae. Vilhelmsen et al. (2010a) provided the first critical phylogenetic evaluation for the group, recognizing Maimetshidae as sister to Trigonalyidae, uniting these two by at least asymmetrical mandibles (not observed in all maimetshids) and the tarsal plantulae in females. Maimetshidae differ from Trigonalyidae, however, at least by the ovipositor exserted (instead of concealed in Trigonalyidae: *vide* Oeser 1962) and the absence of antennal tyloids (these are present in all trigonalyids except the primitive genus *Orthogonalys* Schulz: *vide* Carmean and Kimsey 1998). While the relationship between Maimetshidae and Trigonalyidae now seems well corroborated, the classification of these within an expanded Ceraphronoidea (or Stephanoidea *sensu* Rasnitsyn and Brothers 2009 = Stephanidae, Megalyridae, Trigonalyidae, Maimetshidae, Ceraphronidae, Megaspilidae, Stigmaphronidae, and Radiophronidae) is not well supported, and the phylogenetic placement of Ceraphronoidea s.str. (= Ceraphronidae, Megaspilidae, Stigmaphronidae, and Radiophronidae) is debatable, although many higher-level analyses do recover them as part of the Evaniomorpha (Sharkey 2007; Davis et al. 2010; Vilhelmsen et al. 2010b; Rasnitsyn and Zhang 2010) rather than Proctotrupomorpha (Ronquist et al. 1999; Sharkey and Roy 2002). Most recently Heraty et al. (2011) failed to recover a monophyletic Evaniomorpha, although ceraphronoids did not group with Proctotrupomorpha, but their analysis also had Orussidae nested within a paraphyletic evaniomorph grade which is certainly controversial.

Herein we provide a systematic overview of new material of maimetshids in Cretaceous amber, including the descriptions of three new genera and four new species.

## Material and methods

The species discussed herein were recovered from four different Cretaceous deposits, two in southwestern Europe (France and Spain), one in western Asia (Lebanon), and the last from northcentral North America (Canada). Details of each are briefly summarized below and more thorough discussions on their stratigraphy, paleoecology, and paleoenvironment can be found in Perrichot et al. (2010), Peñalver and Delclòs (2010), Azar et al. (2010), and McKellar and Wolfe (2010), respectively. Morphological terminology generally follows that used elsewhere in the Hymenoptera (e.g., Goulet and Huber 1993), and for consistency the wing terminology as applied in Perrichot et al. (2004a). Acronyms for the institutions with collections studied herein are as follows: MNHN, Muséum National d’Histoire Naturelle, Paris, France; IGR, Géosciences Rennes, Université Rennes 1, Rennes, France; MCNA, Museo de Ciencias Naturales de Álava, Vitoria-Gasteiz, Spain; and CNC-CAS, Canadian National Collection of Insects and Arthropods, Ottawa, Canada.

Photomicrographic images of specimens preserved in transparent amber were prepared using digital cameras attached to stereomicroscopes or to an Infinity K-2 long-distance microscopic lens. Fossils preserved in opaque amber were first detected using propagation phase contrast X-ray synchrotron microradiography, then three-dimensionally imaged using microtomography (PPC-SRµCT), a non-invasive method which allows incomparable possibilities for the visualization of both external and internal structures of amber fossils (Soriano et al. 2010; Perreau and Tafforeau 2011). Imaging was performed on beamline ID 19 of the European Synchrotron Radiation Facility (ESRF) in Grenoble using local tomography protocol (Tafforeau et al. 2006; Lak et al. 2008). The microtomographic scan of specimen IGR.ARC-370.7 was performed using a monochromatic beam at 30 keV with an isotropic voxel size of 5.06 µm and 990 mm of propagation distance. The tomography was obtained using a 180 degree continuous scan with 1500 projections and 0.5s of exposure time. The scan of specimen IGR.ARC-378.2 was performed at 25 keV with an isotropic voxel size of 1.67 µm, 50 mm of propagation distance, and 0.12s of exposure time. After the scan, data were reconstructed using a filtered back-projection algorithm adapted for local tomography applications (PyHST software, ESRF) and adapted ring artifacts correction protocols. Specimen IGR.ARC-370.7 was segmented in 3D using region growing techniques with VGStudioMax (Volume Graphics, Heidelberg, Germany). All of the synchrotron microtomographic data (original stacks of slices, segmentations, animations, pictures, and 3D-volume models) are available at the paleontological online database of the ESRF (http://paleo.esrf.eu), and 3D models in ABS plastic are deposited with the amber piece in Rennes University and at the ESRF.

**French amber.** Since the description of *Guyotemaimetsha enigmatica* by Perrichot et al. (2004a) based on two males, eight new conspecific specimens have been found, including two females previously unknown, five males, and one specimen of uncertain gender. Three further individuals assignable to Maimetshidae may also belong to this species but are too incompletely preserved for accurate attribution.

Six out of the eight new specimens were found in amber from the same outcrop as the type series, i.e., the Font-de-Benon Quarry near the village of Archingeay, in Charente-Maritime, southwestern France. The amber pieces were collected within the lowermost of two amber-bearing strata, e.g., the level A1sl-A *sensu* Batten et al. (2010) = A1sl1 *sensu* Néraudeau et al. (2002). The dating of this level remains problematic, it contains some palynomorphs (dinoflagellate cysts) suggesting a latest Albian age (Dejax and Masure 2005), while recently discovered megaspores are more indicative of the Early Cenomanian (Batten et al. 2010). Two other specimens were collected in a single piece of amber from the same stratigraphic level in the Cadeuil Quarry, about 30 km from Archingeay (see “Arc 1” and “Cdl 1” in Perrichot et al. 2010: figs 1–2).

Similar to the type series, four amber pieces were reworked using a scalpel as a microsaw and the five fossils, separated this way from their syninclusions, were embedded in Canada Balsam between cover slips following the method detailed in Perrichot et al. (2004b). The specimens were assigned collection numbers IGR.ARC-1, IGR.ARC-309.11, IGR.ARC-378.1, IGR.CDL-2.5, and IGR.CDL.2.33. The three other fossils (collection numbers IGR.ARC-370.7, IGR.ARC.378.2, and IGR.ARC-393.4) are preserved in pieces of fully opaque amber and were imaged using PPC-SRµCT as detailed above.

**Spanish amber.** The material originates from Peñacerrada I, in Moraza, Burgos Province, Basque-Cantabrian Basin, Spain (Peñalver and Delclòs 2010). Each fossil is preserved in a thin polished piece of amber, which has been embedded in a clear, synthetic block of epoxy resin according to the method of Corral et al. (1999) except for MCNA-9373, which is not embedded in epoxy. Specimen MCNA-9928, type specimen for one of the new species described herein, is well-preserved and almost complete. Specimen MCNA-13049, holotype for the second species from Spanish amber, is missing the apical parts of the metasoma, of the right antenna, and most legs. Other specimens are incomplete and fossilized in rather opaque amber, partly covered by small bubbles or foggy material, so that some parts are hidden or poorly visible.

**Lebanese amber.** The material originates from an outcrop near Maknouniyyeh Village, Caza (Department) of Jezzine, in southern Lebanon, which corresponds to an ancient field that was used for lignite mining during the French governance of Lebanon and World Wars I and II. The amber is found with lignite in grey lenses of clay corresponding to channels intercalated between fluvial sand deposition of Neocomian age. The precise dating of this sandstone remains problematic and could be anywhere between the Hauterivian and Early Aptian (Azar et al. 2010). The amber is dark orange to red, brittle, with a lot of carbonized vegetal inclusions. This may indicate that the resin was produced in response to a forest fire. The specimen considered herein is exquisitely preserved, missing only the apical portions of the mid and hind legs. It was fossilized in a piece of amber together with a beetle, an evaniid wasp, a roach, and a rhagionid fly, but each inclusion was separated from the others and embedded in Canada balsam between glass coverslips. The specimen is provisionally deposited in the Department of Entomology of the MNHN.

**Canadian amber.** The material was collected from the Taber Coal Zone within the uppermost Foremost Formation, at the Grassy Lake locality in southern Alberta, Canada (McKellar and Wolfe 2010). The specimen was prepared in a similar fashion to the Spanish material described above. The male holotype is nearly complete but is surrounded by a relatively thick layer of dark orange amber with multiple internal fractures; this renders observation difficult. Photographs were taken using glycerin and a cover slip. The type specimen exhibits minor taphonomic distortion, and is missing both metatarsi and much of the forewing apices.

## Systematic paleontology

### Order Hymenoptera Linnaeus, 1758

#### Family Maimetshidae

Rasnitsyn, 1975

http://species-id.net/wiki/Maimetshidae

Maimetshidae Rasnitsyn, 1975: 73. Type genus: *Maimetsha* Rasnitsyn, 1975. Rasnitsyn 1988: 124; Ronquist et al. 1999: 33; Rasnitsyn and Brothers 2009: 192; Perrichot 2009: 2; Ortega-Blanco et al. 2010: 266; Vilhelmsen et al. 2010a: 674.
Dinapsinae [Megalyridae], *partim*: Shaw 1988: 107.
Dinapsini [Megalyridae], *partim*: Shaw 1990: 578.

##### Diagnosis.

Modified from Rasnitsyn and Brothers (2009): Head hypognathous, without ocular carina, without subantennal groove accommodating antennal base; vertex with or without longitudinal median sulcus; antenna filiform, with scape short to moderate, never elongate, flagellomeres variable in number (14–19), most often 16, without apparent sexual dimorphism; mandibles often asymmetrical, with 3–4 teeth. Pronotum short medially; mesoscutum with notauli and median sulcus; axillae meeting anterior to mesoscutellum or separated by scutoscutellar sulcus; propodeum areolate. Forewing with costal space moderate to wide; C and pterostigma present; basal sections of Rs and M subequal in length, not continuously aligned (i.e., not forming smooth basal vein); cell [2R_1_] (i.e., marginal cell) closed, wide (not triangular), moderately short to moderately long; cells [1Rs] and/or [2Rs] closed; cell [1M] closed, small, distant from [1Rs]; cell [2M] open or delimited by spectral or nebulous 2m-cu; 1cu-a antefurcal. Hind wing with no posterobasal lobe; 4–5 distal hamuli; basal cell [R] closed; free apex of Rs present, short to long; free apices of M and Cu short or absent, that of A absent. Legs with trochantelli; tibial spur formula 1-2-2; tarsi pentamerous; tarsal plantulae present in some females; pretarsal claws with preapical tooth. Metasoma rather short, compact, not much sculptured, attached low on propodeum, first segment longest [articulatory ring referred to by Rasnitsyn and Brothers (2009) is not evident and apparently an incorrect interpretation], apical sternum of female elongate, nearly reaching metasomal apex; ovipositor external but not very long, sheaths at most as long as metasoma, not fitting tightly to ovipositor (often preserved detached).

##### Included genera.

*Andyrossia* Rasnitsyn and Jarzembowski, 2000 (Wealden, England, Barremian; a replacement name for *Arossia* Rasnitsyn and Jarzembowski *in* Rasnitsyn et al. 1998); *Ahiromaimetsha* Perrichot, Azar, Nel, and Engel gen n. (Lebanese amber, Neocomian); *Iberomaimetsha* Ortega-Blanco, Perrichot, and Engel gen. n. (Spanish amber, Lower Albian); *Guyotemaimetsha* Perrichot et al., 2004a (French amber, latest Albian/earliest Cenomanian); *Afrapia* Rasnitsyn and Brothers, 2009 (Orapa Mine, Botswana, Turonian); *Afromaimetsha* Rasnitsyn and Brothers, 2009 (Orapa Mine, Botswana, Turonian); *Maimetshorapia* Rasnitsyn and Brothers, 2009 (Orapa Mine, Botswana, Turonian); *Maimetsha* Rasnitsyn, 1975 (Taimyr amber, Santonian); and *Ahstemiam* McKellar and Engel gen. n. (Canadian amber, Campanian). Table 1 summarizes the species diversity of the family.

**Table 1.** Known records for Maimetshidae s.l. (A = amber inclusion; C = compression fossil).

Taxon	Stage	Geography	Sex
Family Maimetshidae Rasnitsyn, 1975			
Genus *Andyrossia* Rasnitsyn & Jarzembowski, 2000			
*Andyrossia joyceae* (Rasnitsyn & Jarzembowski, 1998)	Barremian (C)	England	?
Genus *Ahiromaimetsha* Perrichot, Azar, Nel & Engel, gen. n.			
*Ahiromaimetsha najlae* Perrichot, Azar, Nel & Engel, sp. n.	Neocomian (A)	Lebanon	♀
Genus *Iberomaimetsha* Ortega-Blanco, Perrichot & Engel, gen. n.			
*Iberomaimetsha nihtmara* Ortega-Blanco, Delclòs & Engel, sp. n.	Albian (A)	Spain	♀♂
*Iberomaimetsha rasnitsyni* Ortega-Blanco, Perrichot & Engel, sp. n.	Albian (A)	Spain	♀♂
Genus *Guyotemaimetsha* Perrichot et al., 2004			
*Guyotemaimetsha enigmatica* Perrichot et al., 2004	Albian/Cenomanian (A)	France	♀♂
Genus *Afrapia* Rasnitsyn & Brothers, 2009			
*Afrapia globularis* Rasnitsyn & Brothers, 2009	Turonian (C)	Botswana	♀
*Afrapia variicornis* Rasnitsyn & Brothers, 2009	Turonian (C)	Botswana	♀♂
Genus *Afromaimetsha* Rasnitsyn & Brothers, 2009			
*Afromaimetsha robusta* Rasnitsyn & Brothers, 2009	Turonian (C)	Botswana	♀
Genus *Maimetshorapia* Rasnitsyn & Brothers, 2009			
*Maimetshorapia africana* Rasnitsyn & Brothers, 2009	Turonian (C)	Botswana	♀
Genus *Ahstemiam* McKellar & Engel, gen. n.			
*Ahstemiam cellula* McKellar & Engel, sp. n.	Campanian (A)	Canada	♂
Genus *Maimetsha* Rasnitsyn, 1975			
*Maimetsha arctica* Rasnitsyn, 1975	Santonian (A)	Siberia	♀*

*Incertae sedis* (but possibly maimetshids, *sensu* Rasnitsyn & Brothers 2009)			
Genus *Cretogonalys* Rasnitsyn, 1977**			
*Cretogonalys taimyricus* Rasnitsyn, 1977	Cenomanian (C)	Siberia	?
Genus *Turgonalus* Rasnitsyn, 1990			
*Turgonalus cooperi* Rasnitsyn & Jarzembowski, 1998	Barremian (C)	England	?
*Turgonalus minor* Rasnitsyn, 1990	Neocomian (C)	Siberia	?

* Unfortunately, the holotype of *Maimetsha arctica* was destroyed. Should new material eventually be discovered a neotype will be necessary in order to stabilize the application of the name for the species, genus, and even family. ** *Cretogonalys* is the type genus of the family-group name Cretogonalinae Rasnitsyn, 1977 (*nomen imperfectum*; *recte* Cretogonalyinae Rasnitsyn, 1977), which, if the genus is definitively placed in Maimetshidae, would make the former family-group name a junior synonym of the latter family.

#### Genus Guyotemaimetsha

Perrichot, Nel & Néraudeau, 2004a

http://species-id.net/wiki/Guyotemaimetsha

Guyotemaimetsha Perrichot, Nel & Néraudeau, 2004a: 159 [*Incertae sedis* in Ceraphronoidea*sensu* Rasnitsyn 2002]. Type species: *Guyotemaimetsha enigmatica* Perrichot, Nel & Néraudeau, 2004a. Rasnitsyn and Brothers 2009: 192 [in Maimetshidae]; Ortega-Blanco et al. 2010: 266; Vilhelmsen et al. 2010a: 661.

##### Revised diagnosis.

*Male*: total body length (excluding mandibles and parameres) 1.7 to 2.9 mm; compound eyes oval and bulging, large, 0.66× height of head; vertex without longitudinal medial line; fine occipital carina present; mandibles asymmetrical, right with four teeth and left with three teeth; scape flattened laterally, short, its maximal width 0.9× its length; pedicel nearly as broad as long, 0.75× length of scape; 14 flagellar articles cylindrical in shape and progressively shortening and slightly narrowing; maxillary palpus apparently with five palpomeres; labial palpus apparently with three palpomeres, all short; forewing with pterostigma moderately long, shorter than [2R1]; r-rs originating within apical one-third of pterostigmal length; 2R present as long stub, about half length of 1R; [1M] small, nearly rhombic, with first abscissa of Rs+M one-half length of second abscissa (2Rs+M), so that apical corner (junction of Rs+M and 1m-cu crossvein) positioned anterior to pterostigma; [1Rs] triangular, with Rs and 1rs-m crossvein both fade to become nebulous posteriorly; cross-veins 2rs-m and 2m-cu at most spectral, generally absent; hind wing with cells [R] and [Cu] enclosed by nebulous veins, with free apex of Cu.

*Female*: very similar to male but larger, total body length (excluding mandibles and ovipositor) 3.7 to 4.1 mm; pedicel more elongate, 0.75× as broad as long, 0.85× length of scape; legs with tarsal plantulae; ovipositor apparently simple, not serrate or toothed.

##### Comments.

*Guyotemaimetsha* is most similar to *Maimetsha* in the absence of cell [2Rs]. It differs in cell [1Rs] being anteriorly petiolate instead of sessile, the hind wing with a free apex of Cu, the absence of a medial line on the vertex, and the presence of tarsal plantulae in females.

#### Guyotemaimetsha enigmatica

Perrichot, Nel & Néraudeau, 2004a

http://species-id.net/wiki/Guyotemaimetsha_enigmatica

**Figure 1.**
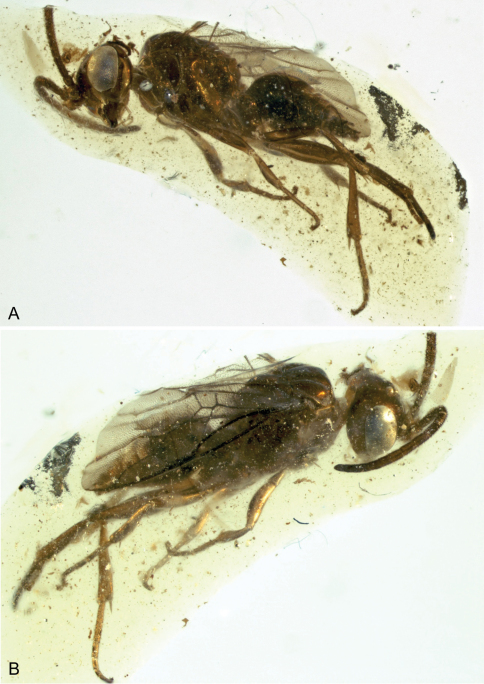
Photomicrographs of male of *Guyotemaimetsha enigmatica* Perrichot et al., 2004a (IGR.CDL-2.5) **A** Left lateral habitus **B** Right lateral habitus.

Figs 1 [Fig F2] [Fig F3] 4

Guyotemaimetsha enigmatica Perrichot, Nel & Néraudeau, 2004a: 159, figs. 1A-B, 2 [♂].

##### Type material.

Holotype MNHN.F.A30174 (♂); paratype MNHN.F.A30175 (♂); in amber collection of the Museum National d’Histoire Naturelle, Paris, France.

##### Other material examined.

Specimens IGR.ARC-1 (♀, missing anterior part of the head and with cuticle somewhat altered by taphonomy), IGR.ARC-309.11 (sex unknown, missing apices of right forewing and metasoma), IGR.ARC-370.7 (= scan ESRF-A-018g, reconstructed, ♀, in opaque amber), IGR.ARC-378.1 (♂, missing head, most metasoma and apices of all legs), IGR.ARC-378.2 (scan ERSF A-401-378, not reconstructed; ♂), IGR.ARC-393.4 (= radiograph ESRF-A-053d, not reconstructed; ♂), IGR.CDL-2.5 (♂, with forewing fragment in IGR.CDL-2.16, missing apical flagellomeres of left antenna; ex collection Arnaud), IGR.CDL-2.33 (♂, missing left antenna and apices of right antenna, right forewing, and all legs; ex collection Arnaud). All specimens deposited in the amber collection of the Department of Geosciences of the University Rennes I, France.

##### Indeterminate specimens.

IGR.ARC-171 (♂, missing head, anterodorsal part of metasoma, and forelegs); IGR.ARC-191 (♂?, missing part of the head and mostly obscured, preserved in very dark amber); IGR.ARC-275 (♀, missing parts of mesosoma, left legs, and with metasoma mostly obscured by a bacterial film). These specimens agree in most respects with the definition of *Guyotemaimetsha enigmatica* but little is preserved and attribution to this species cannot be asserted. All specimens deposited in the amber collection of the Department of Geosciences of the University Rennes I, France.

**Figure 2. F2:**
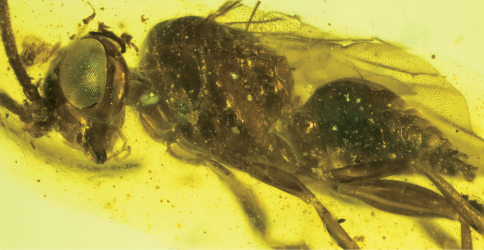
Photomicrograph detail of male of *Guyotemaimetsha enigmatica* Perrichot et al., 2004a (IGR.CDL-2.5).

**Figure 3. F3:**
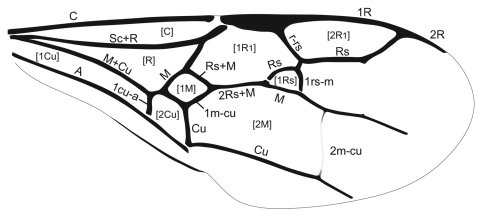
Forewing venation of *Guyotemaimetsha enigmatica* Perrichot et al., 2004a (paratype MNHN-A30175), annotated with vein and cell nomenclature employed herein.

**Figure 4.**
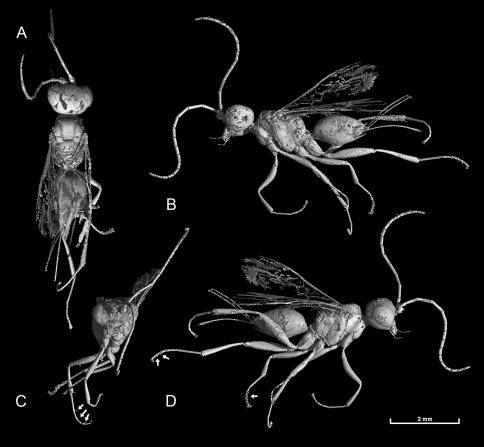
3D virtual extraction of female of *Guyotemaimetsha enigmatica* Perrichot et al., 2004a (IGR.ARC-370.7, scan ESRF-A-018g), using PPC-SRµCT (voxel size 5.06 µm, propagation distance 990 mm, 30 keV) **A** Dorsal habitus **B** Left lateral habitus **C** Facial aspect **D** Right lateral habitus. The arrows indicate tarsal plantulae.

##### Type locality.

Font-de-Benon quarry, 1 km east of Archingeay, Charente-Maritime, France.

##### Stratigraphic horizon.

Lithological subunit A1sl-A, Uppermost Albian–Lowermost Cenomanian, middle Cretaceous.

##### Diagnosis.

As for the genus (see above).

##### Redescription.

*Male*: Integument reddish-brown in color, with dense, minute punctures and fine, decumbent pubescence. Head transverse, with length approximately 0.8x width; ocelli separated from each other by one ocellar diameter; antennae inserted between compound eyes, closer to each other than to compound eye margin or posterior clypeal margin; scape globular in lateral view, laterally compressed in frontal view, with faintly concave apex; pedicel with sides convex; flagellum with dense coat of short, inclined setae, with first article longest, twice length of pedicel; penultimate article as broad as long; apical article with rounded apex; clypeus transverse, about twice as broad as long, with anterior margin rounded and posterior margin straight; mandibles large, with outer margin convex, overlapping apically when closed; right mandible with basal tooth large, second and third teeth equally smaller, apical tooth largest; left mandible with basal tooth large, median tooth smallest, apical tooth largest. Mesosoma compact, 0.7x as high as long; pronotum in dorsal view reduced to a short neck; mesoscutum broader than long, about 0.4x mesosomal length, abruptely truncate anteriorly to form a flat vertical surface, bordered by a transverse carina at anterolateral corners, with low dorsal convexity; median mesoscutal sulcus crenulate, notauli deeply impressed, slightly diverging anteriorly; mesoscutellum with low dorsal convexity, small triangular axillae separated anteriorly by large foveate groove; propodeum areolate, gradually sloping to pronounced posterior lip; meso- and metapleuron with anterior margin foveolate. Forewing hyaline, with costal cell distinctly enlarged around midlength; Rs junction with Sc+R forming an acute angle; cell [1M] with vein M slightly arched, roughly aligned with Rs, vein Cu shortest, M and Rs+M subequal in length; cells [1M] and [1Rs] well distant from each other, separated by long vein 2Rs+M; 2Cu significantly longer than 1Cu and 1m-cu; M and Cu nebulous at apex, reaching wing margin; 2A fading to nebulous vein posterior of [2Cu]. Hind wing with Sc+R, R, M+Cu, Cu, and A tubular, other veins nebulous; Sc+R and R more heavily sclerotized than M+Cu, Cu, and A; Cu fading to nebulous vein apically; free apex of Rs and Cu short. Metacoxa approximately 1.4x length of pro-and mesocoxae, all with broad bases and distinct apical constriction, in lateral view inserted posteroventrally on mesosoma; metafemur distinctly enlarged around midlength; all tibiae approximately subequal to femora in length, with basitarsus length slightly less than combined length of all subsequent tarsomeres. Metasoma compact, as long as mesosoma; parameres trapezoidal, narrowed toward apex, with outer surface slightly convex, with short erect setae around apex; cerci small, spatulate, inserted just anterior to parameres.

*Female*: Very similar to male but with pedicel cylindrical and each leg with a small plantar lobe (or tarsal plantula) on ventral apex of tarsomeres I–IV; ovipositor approximately as long as metasomal length, apex acute. Integument sculpturing and pubescence not visible, altered on specimen IGR.ARC.1 and not reconstructed in microtomographic scan of specimen IGR.ARC-370.7.

#### Iberomaimetsha

Ortega-Blanco, Perrichot & Engel gen. n.

urn:lsid:zoobank.org:act:4AF0B360-B635-4139-B360-FB55DD47179F

http://species-id.net/wiki/Iberomaimetsha

##### Type species.

*Iberomaimetsha rasnitsyni* Ortega-Blanco, Perrichot & Engel, sp. n.

##### Diagnosis.

Antennae with 16 articles; pedicel straight; notauli parallel; forewing costal cell thinner than pterostigma width; prestigma incrassate, wider than base of R, and about the length of 1Rs (distinctly separated from pterostigma); pterostigma longer than distance from base of Rs to base of 2r-rs; cell [2R_1_] short, about 2.4x longer than wide; cells [1Rs] and [2Rs] present but with 2rs-m slightly sclerotized; extremely light nebulous 2m-cu (only visible playing with light incidence angle); protibial spur biseriate apically.

##### Etymology.

The new genus-group name is a combination of Iberia, referring to the Iberian Peninsula, and *Maimetsha*, type genus of the family. The name is feminine.

##### Comments.

*Iberomaimetsha* is well distinguished from *Maimetsha* and *Guyotemaimetsha* in the simultaneous presence of cells [1Rs] and [2Rs] (rm cells *sensu* Rasnitsyn and Brothers 2009); it differs from *Afromaimetsha* by its parallel notauli (instead of diverging anteriorly); the prestigma is incrassate, clearly wider than basalmost R, not as in *Afrapia*; the origin of Rs in *Iberomaimetsha* is well separated from pterostigma, not as close as in *Maimetshorapia*; the pedicel is straight and not “comma-shaped” as in *Ahstemiam* (see below); *Andyrossia* was described from just a forewing but *Iberomaimetsha* differs clearly in several details, such as the length of cell [2R_1_] (around 2.4 times longer than wide versus 3.6 in *Andyrossia*), and the width of cell [C] (narrower than pterostigmal width in *Iberomaimetsha* versus wider in *Andyrossia*).

#### Iberomaimetsha rasnitsyni

Ortega-Blanco, Perrichot & Engel sp. n.

urn:lsid:zoobank.org:act:96249E3D-C395-48CC-BB76-AB746B0F8B4E

http://species-id.net/wiki/Iberomaimetsha_rasnitsyni

Figs 5 [Fig F6] 7

##### Type material.

Holotype MCNA 9928 (♀); paratype MCNA 8765 (♂). All specimens deposited in the Museo de Ciencias Naturales de Álava, Vitoria-Gasteiz, Spain.

##### Type locality.

Peñacerrada I, Moraza, Burgos Province, Basque-Cantabrian Basin, Spain.

##### Stratigraphic horizon.

Escucha Formation, Lower Albian, Lower Cretaceous.

##### Diagnosis.

Mandibles moderately large, each with three teeth, with almost straight margins and teeth (when mandibles opened, teeth facing forward, not curved inward); all flagellomeres of similar length (gradual slight reduction of length); protibial distal margin without comb of stiff setae; tarsal plantulae present on female; cell [1M] about as large as cell [2Cu], both almost square or rhomboidal; 2Rs+M much shorter than 1m-cu and shorter than cell [1Rs]; apicalmost sector of Cu arising at midlength of cell [2Cu]; metasoma elongate, about as long as remainder of body; female ovipositor with apex of second valvula 4-toothed.

##### Description.

**Figure 5.**
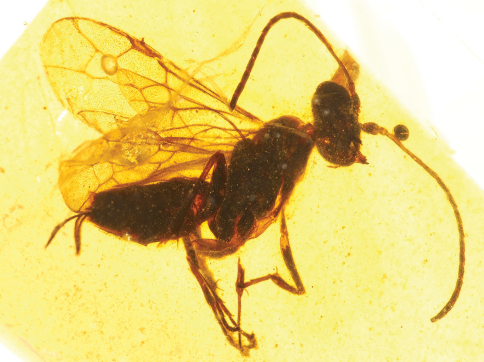
Photomicrograph of holotype female of *Iberomaimetsha rasnitsyni* Ortega-Blanco, Perrichot, and Engel gen. et sp. n. (MCNA 9928).

**Figure 6. F6:**
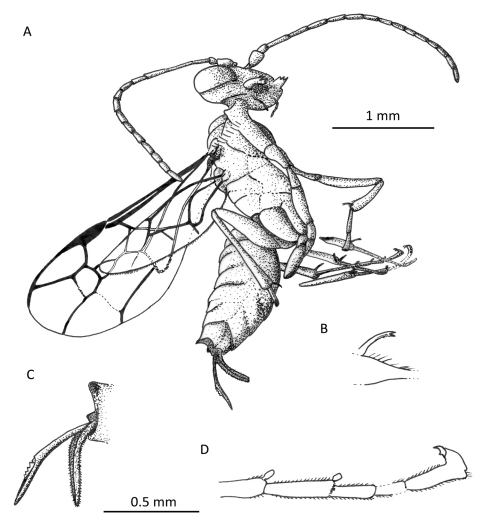
*Iberomaimetsha rasnitsyni* Ortega-Blanco, Perrichot, and Engel gen. et sp. n. (MCNA 9928) **A** Habitus diagram of holotype female **B** Detail of tibial spur **C** Detail of metasomal apex **D** Detail of metatarsus showing tarsal plantulae.

**Figure 7.**
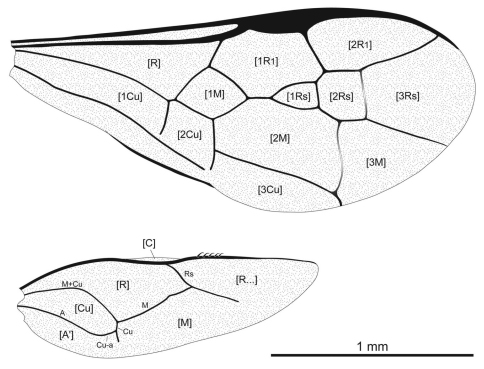
Wing venation of *Iberomaimetsha rasnitsyni* Ortega-Blanco, Perrichot, and Engel gen. et sp. n. (MCNA 9928), annotated with cell nomenclature employed herein.

*Female* (holotype): Body length 2.8 mm. Head rounded in anterior view, length 0.4 mm, width 0.85 mm; compound eyes not especially bulging, oval, large, well separated by 0.43 mm dorsally; basal part of palps not visible but maxillary palps with at least five palpomeres; labial palps with at least three palpomeres; slight, triangular, supra-antennal elevation above torulus, intertorular area flattened; toruli closer to each other than to inner margin of compound eyes; antennae 2.4 mm in length, inserted on frons below midpoint of compound eyes; scape and pedicel short and globular, flagellomeres cylindrical; lengths of antennomeres as follows (all in mm): scape 0.15, pedicel 0.10, flagellomeres 0.22, 0.20, 0.20, 0.20, 0.18, 0.15, 0.14, 0.14, 0.12, 0.12, 0.12, 0.12, 0.12, 0.15; malar space short, 0.15 mm in length. Mesosoma only laterally visible, 1.15 mm in length; pronotum 0.20 mm in length; mesoscutum 0.35 mm in length; mesoscutellum strongly convex, 0.30 mm in length; propodeum slightly rugose, 0.30 mm in length. Only left mid and hind legs are complete, but foreleg can be reconstructed by mixing right and left parts; no visible division of metatrochantellus; tarsi pentamerous, tarsomeres I–IV each with apicoventral plantar lobe (well visible on left metatarsus: Figs 5, 6D); pretarsal claws with small preapical tooth; leg measurements (all in mm): profemur (with protrochantellus) 0.85, protibia 0.50, protarsomeres I–V 0.30/0.15/0.12/?/?; mesofemur 0.75, mesotibia 0.72, mesotarsomeres 0.32/0.15/0.10/0.07/0.12; metafemur 0.95, metatibia 0.90, metatarsomeres 0.38/0.15/0.10/0.08/0.14. Forewing hyaline, length 2.4 mm, maximum width 0.95 mm, densely covered by microtrichia; all veins tubular except for nebulous crossveins 2rs-m and 2m-cu, thus only cells [2Rs] and [2M] pseudo-opened apically (Fig. 7A); 1rs-m not aligned with r-rs; 2m-cu curved at each distal end; veins M and Cu reaching wing margin, with distal end of Cu strongly bent posteriorly; cell [1R_1_] longer than both cells [1Rs] + [2Rs]; cell [1Rs] about half the surface of cells [1M] and [2Rs]; vein r-rs emerging at two-thirds from base of pterostigma; cell [2R_1_] short, with vein Rs slightly anteriorly bent distad 2rs-m. Hind wing hyaline and densely covered by microtrichia, length 1.65 mm, maximum width 0.45 mm; five strong hamuli present on costal vein in distal half of wing (Fig. 7B); distal sections of veins Rs and Cu reaching nebulous wing margin; vein A lost beyond cu-a. Metasoma pedunculate, elongate and globoid, length 1.3 mm, greatest height 0.5 mm; sterna hard, convex; no apparent metasomal armature on sternum II or III; cerci very short; ovipositor moderately exserted, first valvula 0.65 mm long (as preserved), second valvula 0.35 mm long, apically with four dorsal teeth, with sheaths covered by very short, sparse setae (Figs. 4, 5A, 5C).

*Male*: Very similar to female except in following minor differences: Body length 3.63 mm; tarsi without plantar lobes; metasoma slightly longer than mesosoma.

##### Etymology.

The specific epithet is a patronym honoring Prof. Alexandr P. Rasnitsyn for his numerous important contributions to the study of Hymenoptera and his generous friendship with the authors.

#### Iberomaimetsha nihtmara

Ortega-Blanco, Delclòs & Engel sp. n.

urn:lsid:zoobank.org:act:8E3C73D0-4CE1-4910-9DE6-45A0D3BC2F1D

http://species-id.net/wiki/Iberomaimetsha_nihtmara

Figs 8 [Fig F9] 10

##### Type material.

Holotype MCNA 13049 (?); paratypes MCNA 8758(♀?), MCNA 8790 (♂), MCNA 9373 (?), MCNA 9918 (♀), and MCNA 10732 (?). All specimens deposited in the Museo de Ciencias Naturales de Álava, Vitoria-Gasteiz, Spain.

##### Type locality.

Peñacerrada I, Moraza, Burgos Province, Basque-Cantabrian Basin, Spain.

##### Stratigraphic horizon.

Escucha Formation, Lower Cretaceous, Lower Albian.

##### Diagnosis.

Mandibles distinctly large, exserted, asymmetrical, right 4-toothed, left 3-toothed, arched inwards (teeth pointing toward teeth of opposite mandible); basal flagellomeres (from F1 to F3–4) distinctly larger than apical flagellomeres (F1–F4 about 2–1.5× the length of F7–F14); protibial distal margin with a comb of stiff setae; cell [1M] square or rhomboidal, distinctly smaller than [2Cu] which has a short but distinct connection between 1M+Cu and M (then, [2Cu] composed by five aristae); 2Rs+M longer than 1m-cu and longer than cell [1Rs]; apicalmost sector of Cu arising from almost apical posterior margin of [2Cu]; metasoma moderately short, as long as mesosoma.

##### Description.

Body length (from paratypes, as incomplete metasoma in holotype) around 2.55–2.61 mm. Head rounded in anterior view, length 0.41 mm, width 0.68 mm with a short occipital carina and apparently some polygonal dorsal sculpture; compound eyes not bulging, slightly oval, separated dorsally by 0.39 mm; toruli about at same distance to inner margin of compound eyes and each other; antennae 2.54 mm in length, inserted on frons below midpoint of compound eyes; scape and pedicel short and globular, flagellomeres cylindrical; lengths of antennomeres as follows (all in mm): scape 0.12, pedicel 0.09, flagellomeres 0.29, 0.28, 0.27, 0.23, 0.20, 0.18, 0.15, 0.14, 0.12, 0.12, 0.11, 0.11, 0.10, 0.12; malar space short, length approximately 0.11 mm. Mesosoma (dorsal view) 0.95 mm in length; pronotum not distinct dorsally; mesoscutal length 0.27 mm, with parallel notauli and longitudinal medial line, all three present as linear series of grooves more than well-delimited lines (Fig. 9); axillae wide and long, well distinct, separating completely mesoscutellum and mesoscutum; mesoscutellum distinctly convex, length 0.22 mm; propodeum 0.29 mm in length, areolate (seen in paratype MCNA 8790). Legs distinctly elongate; division of trochantellus not visible; tarsi with no plantar lobes visible; leg measurements (all in mm): profemur 0.82, protibia 0.46, protarsomeres I–V 0.34/0.15/0.10/0.09/0.10; mid-legs very poorly preserved but apparently with mesofemur 0.68, mesotibia ?, mesotarsomeres 0.24/0.12/0.10/?/?; metafemur 1.0, metatibia 0.83, metatarsomeres 0.46/0.20/0.15/0.09/0.12. Forewing hyaline, length 2.1 mm, maximum width 0.80 mm, densely covered by microtrichia; all veins tubular except for nebulous 2rs-m and 2m-cu, thus, only cells [2Rs] and [2M] pseudo-opened apically (Figs 9, 10); 1rs-m not aligned with r-rs; 2m-cu curved S-like; veins M and Cu reaching wing margin (although M somewhat nebulous), with apicalmost sector of Cu slightly angled posteriorly; cell [1R_1_] longer than both cells [1Rs]+[2Rs]; cell [1Rs] almost ¾ the surface of cell [1M] and almost half of [2Rs]; vein r-rs emerging at two-thirds from base of pterostigma; cell [2R_1_] short, with vein Rs slightly anteriorly bent distad 2rs-m. Hind wing difficult to observe, hyaline and densely covered by microtrichia, length approximately 1.22 mm, maximum width approximately 0.41 mm; three strong hamuli present on costal vein in distal half of wing. Metasoma (checked on paratype MCNA 8790) pedunculate, short and slightly flattened, length 0.98 mm, greatest height 0.41 mm; sterna hard, convex; no apparent metasomal armature on sternum II or III.

*Male*: Parameres spatulate, with apparently three short stiff setae apically (Fig. 10).

**Figure 8.**
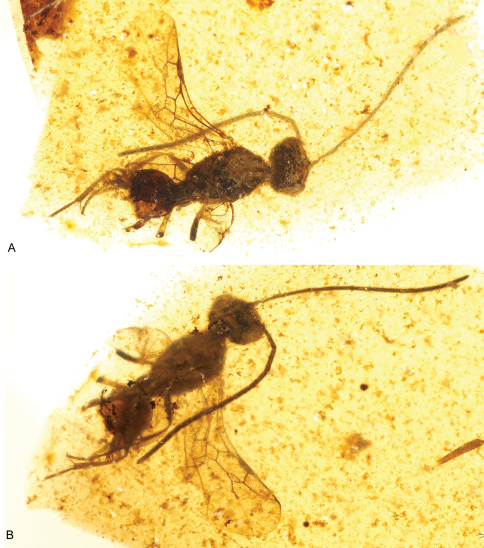
Photomicrographs of holotype of *Iberomaimetsha nihtmara* Ortega-Blanco, Delclòs, and Engel sp. n. (MCNA 13049) **A** Dorsal aspect **B** Ventral aspect.

**Figure 9. F9:**
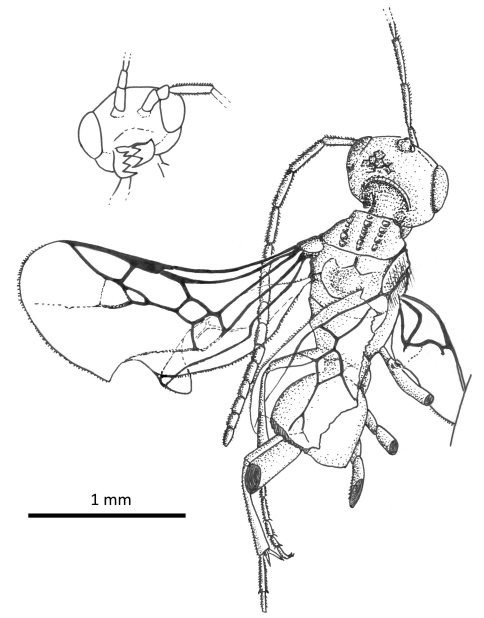
Habitus diagram of holotype of *Iberomaimetsha nihtmara* Ortega-Blanco, Delclòs, and Engel sp. n. (MCNA 13049), with inset of facial aspect.

**Figure 10.**
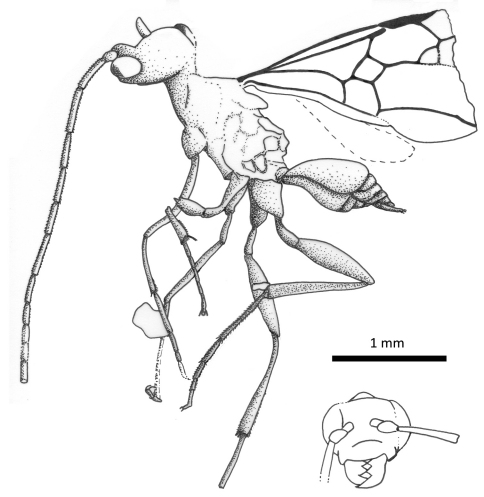
Habitus diagram of male paratype of *Iberomaimetsha nihtmara* Ortega-Blanco, Delclòs, and Engel sp. n. (MCNA 8790), with inset of facial aspect.

##### Etymology.

The specific epithet is a noun in apposition and is the Anglo-Saxon word for nightmare, in reference to the terrifying appearance of the species in frontal view and with its enlarged mandibles.

#### Ahiromaimetsha

Perrichot, Azar, Nel & Engel gen. n.

urn:lsid:zoobank.org:act:56912C39-6C5D-4DF9-81A9-1198DE2C1A6B

http://species-id.net/wiki/Ahiromaimetsha

##### Type species.

*Ahiromaimetsha najlae* Perrichot, Azar, Nel & Engel, sp. n.

##### Diagnosis.

**Figure 11.**
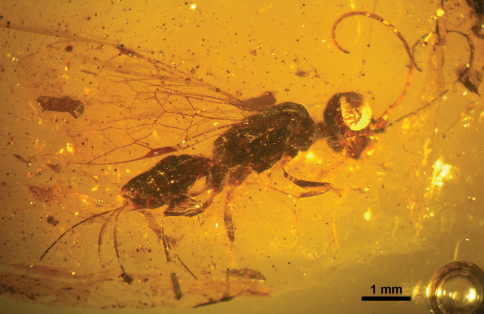
Photomicrograph of holotype female of *Ahiromaimetsha najlae* Perrichot, Azar, Nel, and Engel gen. et sp. n. (MKN-1A).

**Figure 12.**
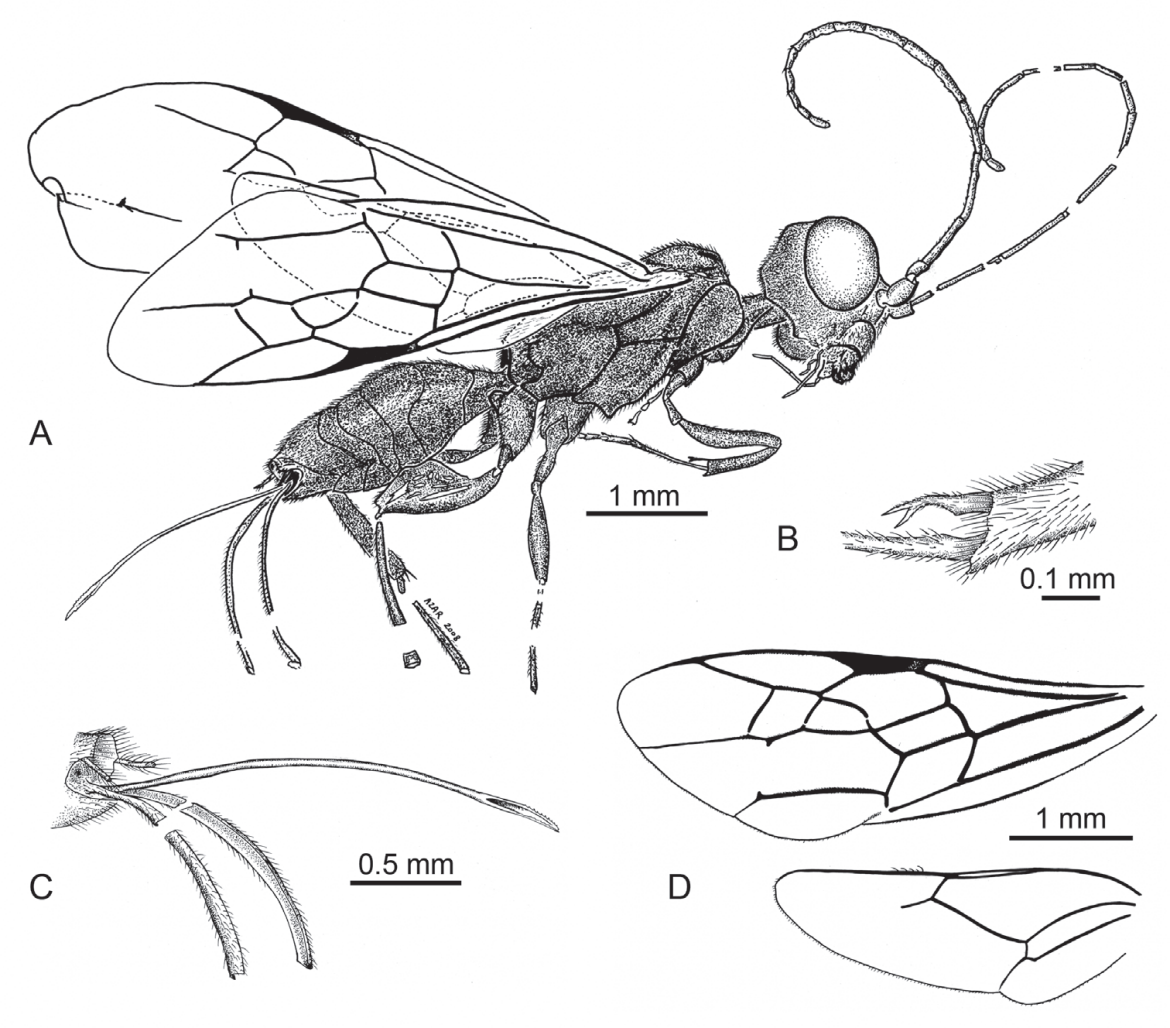
**A** Habitus diagram of holotype female of *Ahiromaimetsha najlae* Perrichot, Azar, Nel, and Engel gen. et sp. n. (MKN-1A), with inset of **B** fore tibial spine **C** ovipositor **D** forewing and hind wing venation.

Antennae apparently with 16 articles; pedicel straight; forewing costal cell apically about as wide as pterostigmal width; prestigma not incrassate, not wider than base of R, about as long as 1Rs (distinctly separated from pterostigma); cell [2R_1_] long, nearly 4x longer than wide; cells [1Rs] and [2Rs] present, former greatly enlarged; 2Rs+M absent owing to confluence of 1m-cu with 2Rs; 1rs-m exceptionally minute (Figs 11, 12D); 2rs-m completely sclerotized; protibial spur bifurcate apically; female tarsi with apicoventral plantar lobes.

##### Etymology.

The new genus-group name is a combination of Ahirom, Phoenician king of Byblos (ca. 1000 BC) whose sarcophagus bears the oldest inscription in the Phoenician alphabet, and *Maimetsha*, type genus of the family. The name is considered to be feminine.

##### Comments.

*Ahiromaimetsha* can be distinguished most easily from other genera by the effective absence of 2Rs+M owing to the confluence of 1m-cu with the second free abscissa of Rs. Like *Iberomaimetsha rasnitsyni* and *Andyrossia joyceae*, *Ahiromaimetsha najlae* has a large cell [1Rs] but even more so than in the aforementioned species and, unlike *Iberomaimetsha rasnitsyni*, the prestigma is not incrassate.

#### Ahiromaimetsha najlae

Perrichot, Azar, Nel & Engel sp. n.

urn:lsid:zoobank.org:act:50E62250-CD58-4CBC-A60F-EEBCBF028F38

http://species-id.net/wiki/Ahiromaimetsha_najlae

Figs 11 12

##### Type material.

Holotype MKN-1A (♀), collection Azar, provisionally deposited in the Muséum National d’Histoire Naturelle, Paris, France.

##### Type locality.

Maknouniyyeh, Caza Jezzine, Mouhafazit Loubnan Al-Janoubi (South Lebanon district), Lebanon.

##### Stratigraphic horizon.

Neocomian (Valanginian–Lowermost Aptian), Lower Cretaceous.

##### Diagnosis.

As for the genus (see above).

##### Description.

*Female*: Total body length ca. 5.2 mm. Head nearly rounded in anterior view, length 0.97 mm, width 1.35 mm, finely setose; compound eyes somewhat bulging, oval, large, length 0.78 mm, width 0.62 mm; ocelli positioned on swelling; mandibles strong, asymmetrical, right with four teeth and left with three teeth both with apical tooth largest; maxillary palps well developed, elongate, distinctly longer than mandibles, with six palpomeres; labial palps short, with four palpomeres; antennae partly damaged but complete, with apparently 16 segments (some segments have secondary constrictions owing to compression), scape and pedicel short and globulous, flagellomeres cylindrical, length of antennomeres as follows (all in mm): scape 0.3, pedicel 0.16, flagellomeres 0.47, 0.52, 0.45, 0.34, 0.32, 0.32, 0.22, 0.17, 0.17, 0.16, 0.16, 0.14, 0.11, 0.11; antenna with fine setae and sparse sensilla. Mesosomal length 2.21 mm, height 1.09 mm, finely setose but without visible foveae or sculpturing; mesoscutal length 0.71 mm, with strong anterior median depression; mesoscutellum not clearly visible, obscured by wings. Metacoxa with an outer basal extension; metatrochantellus diagonally divided and appearing two-segmented; protibial calcar bifurcate (Fig. 12B), with defined velum and trunk, velum smooth, trunk with spine-like microsculpture, basitarsal comb composed of blunt, flattened pegs; pretarsal claws cleft; leg measurements (all in mm, given for those observable): procoxa 0.21, protrochanter 0.42, profemur 0.82, protibia 0.60, probasitarsus 0.61, second protarsomere 0.16, third protarsomere 0.11, mesocoxa 0.39, mesotrochanter 0.55, mesofemur 0.81, metacoxa 0.48, metatrochanter 0.16, metafemur 1.10. Wings hyaline, forewing length 4.81 mm, width 1.54 mm; hind wing length 3.12 mm, width 1.15 mm; venation as in figure 12D; five distal hamuli on hind wing. Metasomal length 2.11 mm; height 0.81 mm; metasomal sterna well sclerotized, convex; ovipositor long, length 2.16 mm, with clearly visible denticles at apex (Fig. 12C).

*Male*: Unknown.

##### Etymology.

The specific epithet is a matronym honoring Dr Najla Zeidane-Gèze, wife of the collector of the holotype.

#### Ahstemiam

McKellar & Engel gen. n.

urn:lsid:zoobank.org:act:C50729F7-36EC-4AD3-BAB9-6CAE13C8D74E

http://species-id.net/wiki/Ahstemiam

##### Type species.

*Ahstemiam cellula* McKellar & Engel, sp. n.

##### Diagnosis.

Total body length near 1.2 mm; compound eyes globular and protuberant; fine occipital carina present; pedicel ‘comma-shaped’ in lateral view, 0.54x length of scape and inserted deeply into scape’s apex; 14 flagellomeres, cylindrical in shape and progressively shortening; maxillary palpus with at least three palpomeres, ultimate palpomere with three apical stiff setae, penultimate palpomere with one apical and two mid-body stiff setae; labial palpus apparently with three palpomeres, all short and bearing numerous stiff setae; junction between Sc+R and Rs very close to pterostigma, 2Sc+R shorter than Rs; pterostigma elongate, longer than [2R_1_], with gradual apical taper; r-rs originating within apical one-third of pterostigmal length; 2R present as small stub; [1M] relatively small, apical corner (junction of Rs+M and 1m-cu crossvein) positioned posterior to pterostigma; [1Rs] triangular, bounded by very thin M posteriorly, 2Rs and 1rs-m forming anterior and apical margins of cell, both fade to become nebulous throughout most of cell’s length; first apparent metasomal segment much shorter than cell [2R_1_].

##### Etymology.

The new genus-group name is the inverse of the type genus, *Maimetsha*, and is considered a meaningless euphonious combination of letters. The name is designated to be feminine in gender.

##### Comments.

*Ahstemiam* is most similar to *Maimetshorapia*. The new genus differs in the presence of a prestigma (abscissa of R between 1Rs and pterostigma) that is only slightly inflated (as opposed to distinctly incrassate) and faded apically, as well as a first apparent metasomal tergum that is much shorter than cell [2R_1_]. The nebulous veins bordering the posterior edges of cell [1Rs], as well as the small size of the cell itself, are distinct among known members of the family Maimetshidae.

#### Ahstemiam cellula

McKellar & Engel sp. n.

urn:lsid:zoobank.org:act:E2504362-00B5-4DF0-8047-2B59E46BBBA0

http://species-id.net/wiki/Ahstemiam_cellula

Figs 13 14

##### Type material.

**Figure 13.**
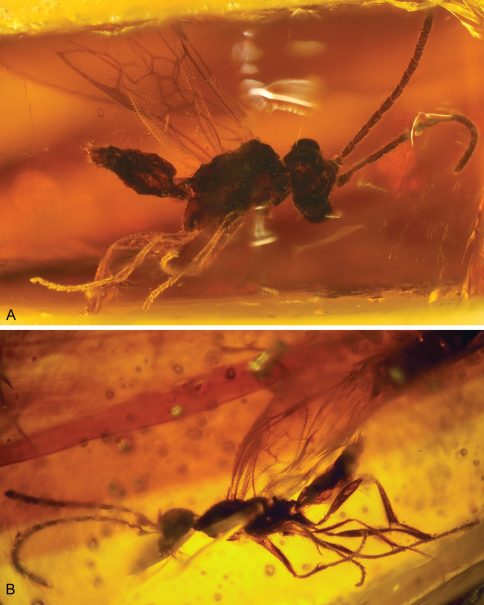
Photomicrographs of holotype male of *Ahstemiam cellula* McKellar and Engel gen. et sp. n. (CNC-CAS 1038) **A** Oblique ventral view **B** Lateral view.

Holotype CNC-CAS 1038 (♂) (Fig. 13). Deposited in Canadian National Collection of Insects and Arthropods, Ottawa, Ontario, Canada.

##### Type locality.

Grassy Lake, Alberta, Canada.

##### Stratigraphic horizon.

Uppermost Foremost Formation, Campanian, Upper Cretaceous.

##### Diagnosis.

As for the genus (see above).

##### Description.

**Figure 14.**
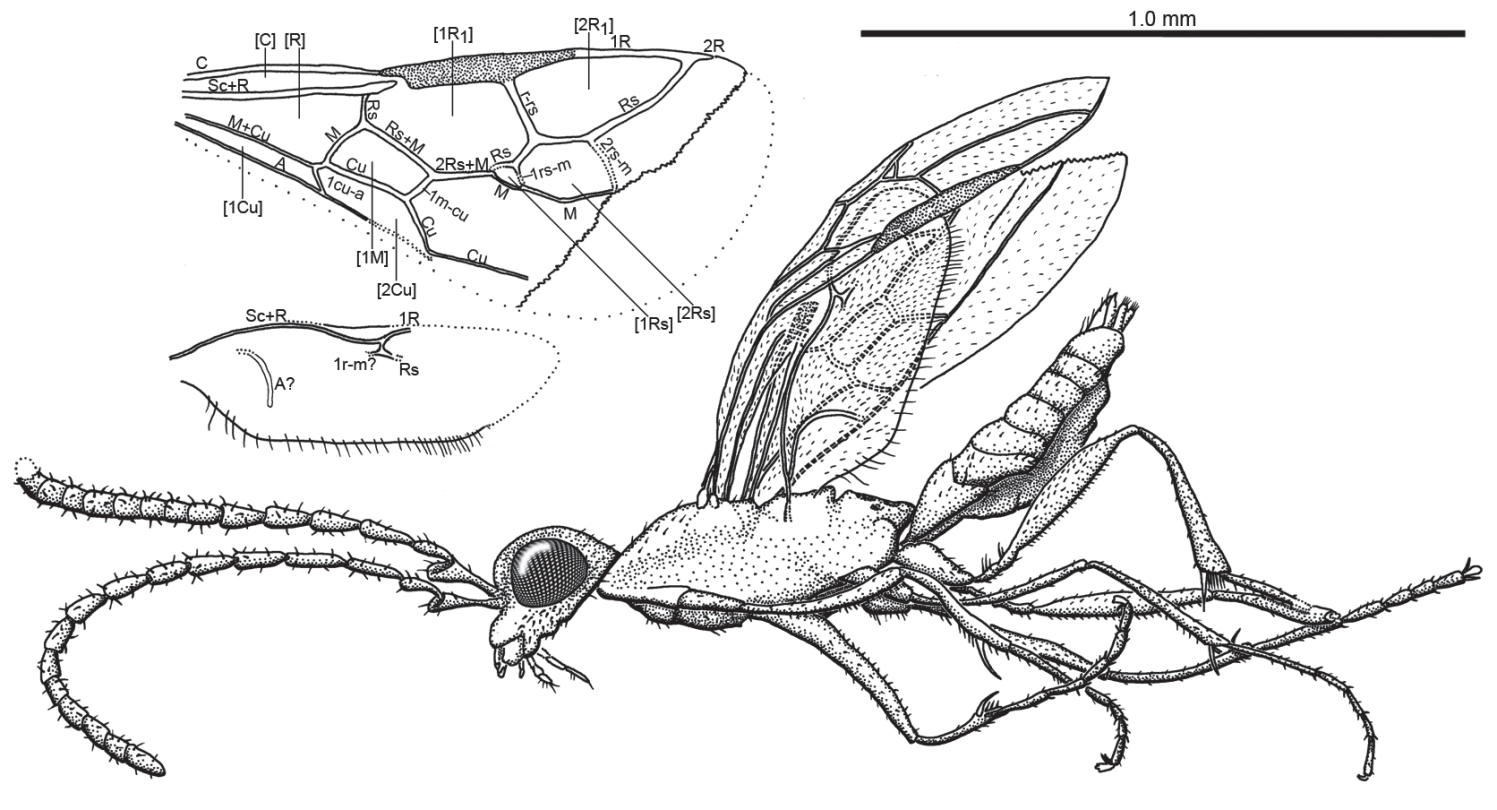
Habitus diagram male of *Ahstemiam cellula* McKellar and Engel gen. et sp. n. (CNC-CAS 1038), with inset of forewing and hind wing venation.

*Male* (holotype): Body color apparently dark brown, with paler brown antennae and femora, and yellow legs distal to femora (Fig. 13). Head slightly distorted in type specimen, appearing broad (transverse) and foreshortened (exsagittal), with length 0.57× width; ventral margin of gena with prominent notches, more so adjacent to mandibles; frons deeply impressed, antennal bases inserted in shallow depression between compound eyes (Fig. 13); vertex with sparse, short, inclined setae; scape taphonomically distorted, in lateral view, elongate and narrow basally with pronounced apical flare, and concave apex (Fig. 14); pedicel with row of midlength stiff setae inclined upon ventral margin; apical flagellar article slightly wider than preceding articles, and tapering to bluntly-rounded apex; flagellum with dense coat of short, curled setae in various orientations. Mesoscutum with low dorsal convexity, apparently near one-half of mesosomal length; mesoscutellum with low dorsal convexity, overhanging metanotum slightly, bearing numerous short, inclined setae; metanotum with prominent ridge along posterior margin which projects posterodorsally over propodeum; propodeum gradually sloping to pronounced posterior lip. Forewing at least 0.96 mm in length and 0.42 mm in width, hyaline with microtrichial vestiture and pterostigma pale brown, like tubular veins in wing; Sc+R slightly thicker than other veins, narrowing basally but expanding apical to junction with Rs; fusion of Rs and M angled, veins not parallel to each other; 2rs-m apparently nebulous throughout its course, but preserved in only one wing, which appears taphonomically affected in this region; apex of M not preserved in type specimen; 2Cu significantly longer than 1m-cu; 3Cu appears relatively short, terminating posterior to 1rs-m; 2A fading to nebulous vein at midlength of cell [2Cu]. Hind wings poorly visible; apparent length near 0.64 mm; only anterior veins partly visible; broad, open costal cell apparent near apex of Sc+R; R appears to curve anteriorly and rapidly fade along anterior margin of wing; 2Rs and putative 1rs-m both fade distally, and are removed from 1R by relatively long 1Rs; faint curved vein within posterior portion of wing base assumed to represent A; marginal setae visible along posterior margin of wing are fine, elongate and relatively sparse, with greater density in apical positions. Legs with pervasive coat of short, stiff setae densely inclined upon most surfaces; pro- and mesocoxae with elongate setae inclined upon ventral surface; profemur with two rows of short, stiff setae suberect upon ventral surface, protibia bearing row of at least three stout spines approximately one-half of calcar’s length on posterior surface; calcar gently curved and robust (as wide as probasitarsus), deeply inserted into protibial apex, extending only slightly beyond apex; fine setae inclined upon both sides of calcar, and matching longitudinal comb of erect spicules on basal half of probasitarsus; probasitarsus length slightly less than all subsequent tarsomeres combined, with approximately four irregularly distributed spines upon apical half of plantar surface; protarsomeres III and IV much shorter than others, one-half to two-thirds length of protarsomere V; all tarsi with serrate pretarsal claws and large arolium, with two apical spines on all tarsomeres (potentially absent on protarsomeres, but orientation obscures observation); meso- and metacoxae both approximately 1.5× length of procoxa, with broad bases and apical constriction; mesofemur with two rows of short, stiff setae inclined upon ventral surface; mesotibia with patch of fine, short spines along posterior surface near apex; two mesotibial spurs short and stout, slightly longer than apical width of mesotibia, and bearing minute setae along both margins; metafemur with significant midlength inflation, laterally compressed, with two rows of short, stiff setae suberect along ventral surface; metatibia with moderate apical expansion, bearing two apical spurs slightly longer than metatibial apical width, as well as comb-like row of six spicules each approximately two-thirds of spurs’ length; metatarsi not preserved. Metasoma with six apparent terga; terga appear to bear frills of elongate setae along posterior and lateral margins, terga more pilose within posterior of metasoma; parameres spatulate, each with four elongate setae erect on apex.

*Female*: Unknown.

##### Etymology.

The specific epithet is the Latin diminutive noun in apposition, *cellula* (meaning, “small room”) and refers to the very small size of cell [1Rs].

### Key to genera and species of Maimetshidae

The following key is modified from that of Rasnitsyn and Brothers (2009).

**Table:** 

1	Forewing with three submarginal cells, i.e. cells [1Rs] and [2Rs] both present, [1Rs] sometimes incompletely delimited (Fig. 7); flagellomere number 14–19 (unknown in *Andyrossia* Rasnitsyn and Jarzembowski)	2
–	Forewing with two submarginal cells, i.e. cells [1Rs] and [2Rs] not simultaneously present (Fig. 3); 14 flagellomeres	10
2(1)	Forewing 2Rs+M (second abscissa Rs+M, distal to 1m-cu) shorter than 1m-cu to effectively absent (Figs 7, 12D)	3
–	Forewing 2Rs+M (second abscissa Rs+M, distal to 1m-cu) subequal to or longer than 1m-cu (Figs 9, 10, 14)	5
3(2)	Forewing 1m-cu basad to second free abscissa of Rs such that 2Rs+M present (Fig. 7; Rasnitsyn et al. 1998: fig. 13)	4
–	Forewing 1m-cu effectively confluent with second free abscissa of Rs such that 2Rs+M absent (Figs 11, 12) (Neocomian, Lebanon)	*Ahiromaimetsha najlae* Perrichot, Azar, Nel & Engel, gen. et sp. n.
4(3)	Forewing cell [2Rs] not widened anteriorly; prestigma incrassate, about as long as 1Rs; 2m-cu present; pterostigma slightly widening apically (Figs 5–7) (Albian, Spain)	*Iberomaimetsha rasnitsyni* Ortega-Blanco, Perrichot & Engel, gen. et sp. n.
–	Forewing cell [2Rs] widened anteriorly; prestigma linear, longer than 1Rs; 2m-cu absent; pterostigma linear (Rasnitsyn et al. 1998: fig. 13) (Barremian, England)	*Andyrossia joyceae* (Rasnitsyn and Jarzembowski)
5(2)	Forewing cell [1Rs] not reduced, 1rs-m longer than 3Rs (abscissa of Rs distad 1rs-m; Fig. 10); pedicel, where known, not curved	6
–	Forewing cell [1Rs] greatly reduced, 1rs-m much shorter than 3Rs (abscissa of Rs distad 1rs-m); pedicel arched (Figs 13, 14) (Campanian, Canada)	*Ahstemiam cellula* McKellar & Engel gen. et sp. n.
6(5)	Notauli more or less parallel (Fig. 9)	7
–	Notauli strongly diverging anteriorly (Rasnitsyn and Brothers 2009: fig. 7b) (Turonian, Botswana)	*Afromaimetsha robusta* Rasnitsyn & Brothers
7(6)	Forewing Rs originating well before pterostigma, prestigma about as long as or longer than 1Rs (Figs 9, 10)	8
–	Forewing Rs originating close to pterostigma, prestigma much shorter than 1Rs; prestigma incrassate, swollen apically and broader than basal abscissa Rs (Rasnitsyn and Brothers 2009: fig. 8b) (Turonian, Botswana)	*Maimetshorapia africana* Rasnitsyn & Brothers
8(7)	Prestigma linear and similar to basal abscissa R (Rasnitsyn and Brothers 2009: figs 1, 2); 2m-cu absent (Genus *Afrapia* Rasnitsyn and Brothers)	9
–	Prestigma incrassate; 2m-cu present, nebulous (Figs 8, 9) (Albian, Spain)	*Iberomaimetsha nihtmara* Ortega-Blanco, Delclòs & Engel, sp. n.
9(8)	Pterostigma shorter than (about 0.8x) distance between base of Rs and 2r-rs (Rasnitsyn and Brothers 2009: fig. 1b) (Turonian, Botswana)	*Afrapia globularis* Rasnitsyn & Brothers
–	Pterostigma as long as or longer than distance between base of Rs and 2r-rs (Rasnitsyn and Brothers 2009: fig. 2b) (Turonian, Botswana)	*Afrapia variicornis* Rasnitsyn & Brothers
10(1)	Forewing cell [1Rs] anteriorly petiolate; hind wing with free apex of Cu; vertex without medial longitudinal line; compound eyes bulging; tarsal plantulae present in females (Figs 1–4) (latest Albian/earliest Cenomanian, France)	*Guyotemaimetsha enigmatica* Perrichot et al.
–	Forewing cell [1Rs] anteriorly sessile; hind wing without free apex of Cu; vertex with medial longitudinal line; compound eyes not bulging, almost following head contour; tarsal plantulae absent (Rasnitsyn 1975: fig. 87, pl. IV, figs. 14a, 14b) (Santonian, Siberia)	*Maimetsha arctica* Rasnitsyn

## Discussion

The new specimen from Canadian amber extends the temporal range of the family into the Campanian (from the Barremian to the Campanian), as the previous youngest exemplar was *Maimetsha*, from the Santonian amber of Taimyr (Siberia). Our new record also extends the palaeobiogeographic distribution of the family to encompass North America (from south Gondwana to north Laurentia), suggesting an origin during the Early Mesozoic, perhaps sometime in the mid-Jurassic.

Certainly much remains to be undertaken on Maimetshidae, and research into the family is in its infancy. It will be exciting to develop eventually a broad data set meant to examine the interrelationships among the species (indeed, even addressing maimetshid monophyly!) as well along with their relatives among the Trigonalyidae and Megalyridae (along the lines of the study of Vilhelmsen et al. 2010a), and perhaps also among the Ceraphronoidea s.str. In addition, it is not entirely clear that those specimens known only from wing venation are definitively maimetshids, or alternatively, what other fragmentary taxa previously ascribed to Trigonalyidae or elsewhere might more accurately belong in or near Maimetshidae. Once a thorough cladistic analysis has been undertaken for maimetshids, preferably after a greater wealth of diversity and material is discovered and more species analyzed using modern tools such as synchrotron imaging, then the generic classification of the family should be re-evaluated as it presently appears somewhat finely divided.

For now, what started as a singular species discovered and presciently interpreted by Alex Rasnitsyn 36 years ago (Rasnitsyn 1975), has grown rapidly in diversity within the last six years. We can only hope that this trend will continue in the years to come.

## Supplementary Material

XML Treatment for Maimetshidae

XML Treatment for Guyotemaimetsha

XML Treatment for Guyotemaimetsha enigmatica

XML Treatment for Iberomaimetsha

XML Treatment for Iberomaimetsha rasnitsyni

XML Treatment for Iberomaimetsha nihtmara

XML Treatment for Ahiromaimetsha

XML Treatment for Ahiromaimetsha najlae

XML Treatment for Ahstemiam

XML Treatment for Ahstemiam cellula
